# Arterial Doppler Waveforms Are Independently Associated With Maximal Walking Distance in Suspected Peripheral Artery Disease Patients

**DOI:** 10.3389/fcvm.2021.608008

**Published:** 2021-04-20

**Authors:** Annaïg Miossec, Quentin Tollenaere, Damien Lanéelle, Antoine Guilcher, Antoine Métairie, Estelle Le Pabic, Awenig Carel, Alexis Le Faucheur, Guillaume Mahé

**Affiliations:** ^1^Vascular Medicine Unit, CHU Rennes, Rennes, France; ^2^Vascular Medicine Unit, CHU Caen Normandie, Caen, France; ^3^CHU Rennes, Inserm, CIC 1414 (Clinical Investigation Center), Rennes, France; ^4^Univ Rennes, M2S-EA 7470, Rennes, France; ^5^Univ Rennes 1, Rennes, France

**Keywords:** Doppler waveforms, peripheral artery disease, maximal walking distance, ankle-, brachial index, peripheral artery (occlusive) disease

## Abstract

**Objective:** Arterial Doppler waveform recordings are commonly used to assess lower extremity arterial disease (LEAD) severity. However, little is known about the relationship between arterial Doppler waveform profiles and patients' walking capacity. The purpose of this study was to assess whether arterial Doppler waveforms are independently associated with maximal walking distance (MWD) in patients experiencing exertional limb symptoms.

**Materials and Methods:** This cross-sectional study included suspected LEAD patients experiencing exertional limb symptoms. In both lower extremities, arterial Doppler waveforms and ankle-brachial index (ABI) values were obtained from the pedis and tibial posterior arteries. Each arterial flow measurement was ranked using the Saint-Bonnet classification system. Treadmill stress testing (3.2 km/h, 10% slope) coupled with exercise oximetry (Exercise-TcPO2) were used to determine MWD. Delta from rest oxygen pressure (DROP) was calculated. Following treadmill stress testing, post-exercise ABI values were recorded. Univariate and multivariate analyses were used to determine the clinical variables associated with MWD.

**Results:** 186 patients experiencing exertional limb symptoms (62 ± 12 years and 26.8 ± 4.5 kg/m^2^) were included between May 2016 and June 2019. Median [25th; 75th] treadmill MWD was 235 [125;500]m. Better arterial Doppler waveforms were associated with better walking distance (*p* = 0.0012). Whereas, median MWD was 524 [185;525]m in the group that yielded the best Doppler waveforms, it was 182 [125,305]m in the group with the poorest Doppler waveforms (*p* = 0.0012). MWD was significantly better (*p* = 0.006) in the patients with the best ABIs. However, arterial Doppler waveforms alone were significantly associated with MWD (*p* = 0.0009) in the multivariate model. When exercise variables (post-exercise ABI or DROP) were incorporated into the multivariate model, these were the only variables to be associated with MWD.

**Conclusion:** Of the various clinical parameters at rest, Doppler flow waveform profiles were associated with MWD in suspected LEAD patients. A stronger link was however found between exercise variables and MWD.

## Introduction

Lower extremity arterial disease (LEAD) is a common condition that affects an estimated total of over 230 million patients worldwide (1). Half of these patients experience exertional limb symptoms resulting in a negative impact on quality of life (2).

Ankle-brachial pressure index (ABI) at rest is one means of diagnosing LEAD, whereby values equal to or <0.90 are regarded as pathological (3, 4). Lower resting ABI values are also known to be associated with more severe LEAD (5, 6). Several studies have assessed an association between ABI and walking impairment but the results remain controversial (7–11). There are three main explanations for the disparity found: (i) the small sample size of included patients ranging from 14 to 156; (ii) the lack of homogeneity among the populations of interest; and (iii) the variety of testing protocols employed. Indeed, Gardner and colleagues (8) reported correlation between ABI and maximal walking distance (MWD) when assessed according to a graded testing protocol (speed 2 mph at 0% gradient increased by 2% every 2 min) but not when a single-stage protocol (1.5 mph and 7.5% gradient) was employed (8).

Arterial Doppler waveform recordings are commonly used for the purposes of LEAD diagnosis and follow-up (12–14). Arterial Doppler waveforms are influenced by the extent of upstream arterial stenosis (13, 15). Thus, arterial Doppler waveform profiles provide useful information, especially in medial calcific sclerosis patients whose ABI is unreliable (12, 16), as they may be indicative of LEAD severity. As such, walking impairment in LEAD patients could be linked to Doppler waveform profiles. To date, no study has addressed this issue.

If clinical signs or bedside assessments were able to determine functional limitations of patients, this would be of great interest to clinicians insofar as treadmill tests and 6-min walk tests would prove unnecessary.

The aim of this study was therefore to assess the relationship between Doppler waveform profiles and maximal walking distance in: (i) patients experiencing exertional limb symptoms; and (ii) in and confirmed LEAD patients experiencing exertional limb symptoms.

## Methods

### Ethical Considerations

The present study was conducted from May 2016 to June 2019 and received institutional review board approval (IRB; no. 17.12). All of the participants provided written informed consent. The study protocol is consistent with 1975 Declaration of Helsinki ethical guidelines. The “Exercise PAD” study was registered with the American National Institute of Health database under reference number NCT03186391.

The data substantiating the findings from this study are available from the corresponding author upon reasonable request. The corresponding author had full access to all of the study data and takes responsibility for its integrity and for all data analysis.

### Study Design

This cross-sectional single-institution study included consecutive patients referred to the vascular unit of Rennes University Hospital (France) for exertional limb symptoms suggestive of LEAD. The relationship between Doppler waveform profiles and maximal walking distance was assessed in: (i) patients experiencing exertional limb symptoms (suspected LEAD); and (ii) patients experiencing exertional limb symptoms AND at least one hemodynamic criterion for LEAD (confirmed LEAD).

### Inclusion and Exclusion Criteria

Patients were included provided they met all of the following criteria:

- clinical history of exertional limb symptoms suggestive of LEAD (i.e., pain in active muscle groups occurring in the course of exercise and lasting for under 10 min);
- arterial Doppler waveform data availability for both limbs;
- ABI data availability for both limbs;
- completion of treadmill exercise testing at 3.2 km/h and 10% slope;
- written informed consent.

Patients were excluded if they met at least one the following criteria:

- treadmill exercise testing discontinued on account of symptoms other than ischemia-induced lower-limb pain (e.g., dyspnea, angina, rheumatic pain)
- incomplete resting ABI and arterial Doppler waveform data.

### Characteristics of Patients

Collected variables included age, sex, body mass index, smoking status, comorbidities, and medication (statins, anti-hypertensive treatment, antiplatelets, oral antidiabetic treatment or insulin). Patients were classified as confirmed LEAD if they fulfilled at least one of the following criteria: ABI ≤ 0.90 or Doppler waveform profile other than N or A under the simplified Saint-Bonnet classification system (see below); or a decrease in post-exercise ABI ≥18.5% (see below) or delta from rest oxygen pressure (DROP) ≤-15 mmHg (see below).

### ABI Measurement

ABIs were measured by a trained vascular medicine physician (4) consistent with American Heart Association guidelines using a hand-held Doppler probe (8 MHz; Basic Atys Medical, Soucieu en Jarrest, France), with the exception of brachial blood pressure that was measured using an automated oscillometric blood pressure monitor (Carescape Dinamap V100; GE Healthcare) (17). Measurements were taken after a 10-min supine rest period, head and heels supported, in a temperature-controlled room (21°C) (18). The following counterclockwise sequence was used: right brachial artery, right posterior tibial artery, right dorsalis pedis artery, left posterior tibial artery, left dorsalis pedis artery, left brachial artery, and right brachial artery. The ABI was calculated by dividing the highest lower limb pressure (dorsalis pedis or posterior tibial pressures) by the highest arm pressure as recommended (4, 18). The symptomatic lower limb ABI as reported via case history was used for the purposes of analysis.

### Arterial Doppler Waveform Recordings

**Figure 1:**
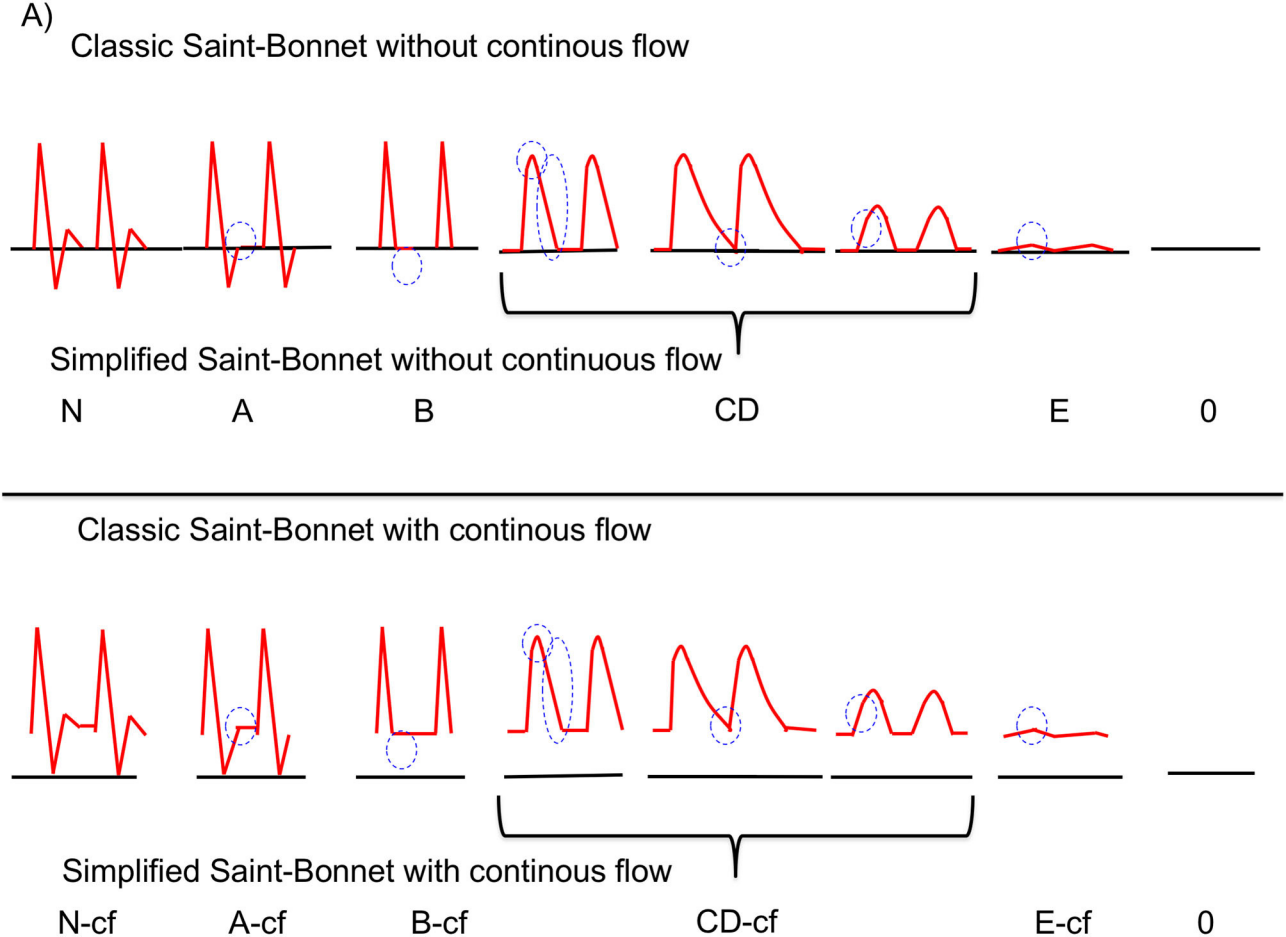
Simplified Saint-Bonnet Classification. Saint-Bonnet classification according to arterial lesion severity (from type N to 0). cf, continuous flow. The blue circles represent the key elements that vary from type to type. Of note: continuous flow may be horizontal or slightly descending. This diagram shows stable continuous flow and is therefore depicted horizontally. For more information please refer to initial Vasa publication (13).

Arterial Doppler waveforms were acquired in both lower extremities from the pedis and tibial posterior arteries before ABI measurement. Each arterial flow measurement was ranked using the Saint-Bonnet classification system as recommended in France by professors from the French College of Vascular Medicine (CEMV) (13, 19). In brief, the simplified version of this classification system portrays types according to arterial lesion severity (Saint-Bonnet N, Saint-Bonnet A, Saint-Bonnet B, Saint-Bonnet CD, Saint-Bonnet E, Saint-Bonnet 0 [i.e., no flow]) (13, 19). The Saint-Bonnet classification system is outlined in Figure 1. Saint-Bonnet N and A are regarded as normal whereas any other type of flow is regarded as symptomatic of LEAD. Only the Doppler waveforms from the pedis and tibial posterior arteries of the symptomatic limb reported via case history were used for analysis. Doppler flow waveforms from the pedis and tibial posterior arteries were ranked in accordance with the Saint-Bonnet classification system. The waveforms ranked closest to N were labeled the “best waveforms” and those ranked closest to E or 0 were labeled the “worst waveforms.” Each limb was thus characterized by two flows: a best flow and a worst flow.

### Treadmill Stress Testing Coupled With Exercise Transcutaneous Oxygen Pressure Measurement (Exercise-TcPO2)

A treadmill stress test (3.2 km/h, 10% slope) was used to determine MWD (17). The patients were asked to inform the physician of pain onset (17). Exercise was discontinued when patients reached their thresholds or lasted for a maximal distance of 525 m (over a period of 10 min) (17).

Exercise-TcPO2 was measured during the treadmill stress test as previously documented using calibrated TcPO2 electrodes (TCOM/TcPO2; PF 6000TcPO2/CO2 Unit; Perimed^®^ Jarfalla, Sweden) (20–23). Delta from rest oxygen pressure (DROP) was recorded in each lower extremity in real time by in-house validated Oxymonitor (version 2019.01.05) free software (https://imagemed.univ-rennes1.fr/en/oxymonitor/download) as previously documented (17, 24). As defined in previous studies, DROP ≤-15 mmHg was used to diagnose LEAD (17, 20, 22).

### Post-exercise ABI

Readings were taken by two vascular physicians: one reading from the brachial artery using an automatic blood pressure device (Carescape Dinamap V100; GE Healthcare) and one from the lower limb with a handheld Doppler device. Post-exercise brachial pressure was taken from the same artery that was used for ABI measurement at rest. While resting ABI was being measured, a black pen was used to mark the skin area in which the highest lower limb pressure had been recorded by a hand-held Doppler device (25, 26) in order to streamline the post-exercise measurement procedure. As defined in a previous study, a post-exercise decrease in ABI ≥18.5% was used to diagnose LEAD (17).

### Statistical Analysis

#### Data Analysis

Continuous variables were expressed as means ± standard deviation (sd) regarding normal distribution and as median and interquartile range (IQR) values regarding non-normal distribution, and categorical variables were expressed as counts (percentages). With respect to quantitative variables, the Student's *t*-test or U Mann-Whitney test were used to compare baseline characteristics where two groups were involved and the one-way Anova and Kruskal-Wallis tests where more than two groups were involved; the chi-squared test or Fisher's exact test were used for qualitative variables, subject to distribution. Analysis was performed per patient, rather than per limb and only the symptomatic lower limb reported via case history was included in the analysis. Where both lower limbs were symptomatic, the symptomatic lower limb was selected randomly. In our experience of analyzing of Doppler waveforms, the prevalence of E and 0 Doppler flow waveforms as per Saint-Bonnet classification is low. Thus, Doppler flow waveforms categorized as CD, E or 0 were placed in the same category, in effect reducing the number of flow waveform categories to 4 (N, A, B and CDE0). First and foremost, univariate logistic regression was conducted to define the variables representing the characteristics of patients and at-rest clinical variables (ABI and Doppler waveforms) associated with MWD in the suspected LEAD population in the first instance and in the confirmed LEAD population in the second. Then variables whose *p*-value was <0.20 were included in multivariate analysis. A backward stepwise procedure was used. A significance threshold of 0.05 was used for all statistical testing. Finally, we incorporated exercise variables (post-exercise ABI and DROP) into the model to assess whether there was a stronger correlation between these variables and MWD in both populations. Statistical analysis was performed using SAS 9.4 software (SAS Institute, Cary, NC, USA) (https://www.sas.com/en_us/home.html).

## Results

### Population of Interest

**Figure 2:**
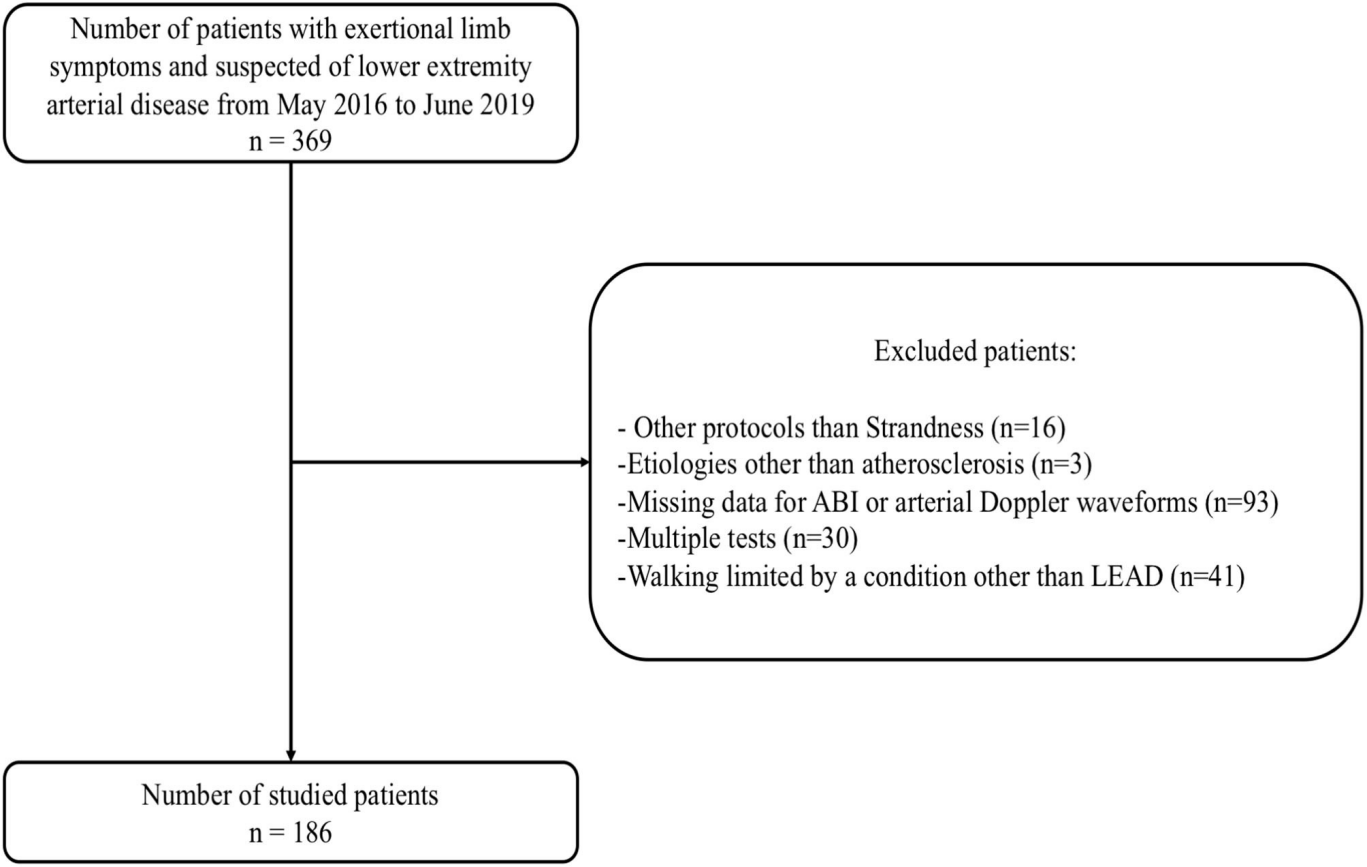
Flowchart of included population.

Of the 369 consecutive patients who attended the clinic for standard care, 186 patients with exertional limb symptoms were included (Figure 2). The reasons for exclusion and the corresponding proportion of potential participants meeting each criterion were as follows: protocol other than Strandness with a 10% slope and 3.2 km/h (*n* = 16; 9%); etiologies other than LEAD atherosclerosis (*n* = 3; 2%); missing ABI or arterial Doppler waveforms data (*n* = 93; 51%); walking hampered by a condition other than LEAD (*n* = 41; 22%); and multiple tests (*n* = 30; 16%).

Characteristics of patients are listed in Table 1. Mean age and body mass index were 62 ± 12 years and 26.8 ± 4.5 kg/m^2^, respectively. Median treadmill MWD was 235 [133–500]m. Eighteen patients fully completed the treadmill stress test. Patient totals in each Saint-Bonnet category were 21 (11%), 67 (36%), 44 (24%), 52 (28%), 2 (1%), and 0 (0%) pertaining to Saint-Bonnet N, Saint-Bonnet A, Saint-Bonnet B, Saint-Bonnet CD, Saint-Bonnet E, and Saint-Bonnet 0 categories, respectively.

**Table 1:** Population characteristics.

**Clinical characteristics**	**Suspected LEAD population** **(*n* = 186)**	LEAD population with at least one criterion^*^ **(*n* = 165)**
Men (%)	150 (81%)	137 (83%)
Age in years (mean ± SD)	62 ± 12	63 ± 10
Body mass index, kg/m^2^ (mean ± SD)	26.8 ± 4.5	26.8 ± 4.6
Comorbidities, (history of), no. (%)		
Smoker (current or former)	152 (84%)	134 (84%)
Coronary disease	58 (33%)	56 (36%)
Hypercholesterolemia	127 (68%)	119 (72%)
Diabetes	38 (20%)	38 (23%)
Chronic kidney disease	10 (5%)	10 (6%)
Hypertension	129 (69%)	122 (74%)
Stroke (ischemic stroke; transient ischemic attack)	19 (11%)	19 (12%)
Current medications, no. (%)		
Statins	118 (63%)	112 (68%)
Anticoagulants	20 (11%)	19 (12%)
Antiplatelets	147 (79%)	139 (84%)
Other anticholesterolaemic drugs	16 (9%)	15 (9%)
Angiotensin-converting enzyme inhibitors	78 (42%)	74 (45%)
Ankle brachial index at rest (right)	0.88 ± 0.29	0.85 ± 0.30
Ankle brachial index at rest (left)	0.88 ± 0.28	0.85 ± 0.26
Maximal treadmill walking distance, m	235 [133–500]	226 [126–407]

*Results are shown as mean ± standard deviation or medians [Quartile 1; Quartile 3], or number of observations (%)*.

* *Criteria for LEAD were ABI ≤0.90 or abnormal Doppler waveforms or post-exercise ABI decrease ≥18.5% or DROP ≤-15 mmHg*.

### Relationship Between MWD and Arterial Doppler Waveforms

**Figure 3:**
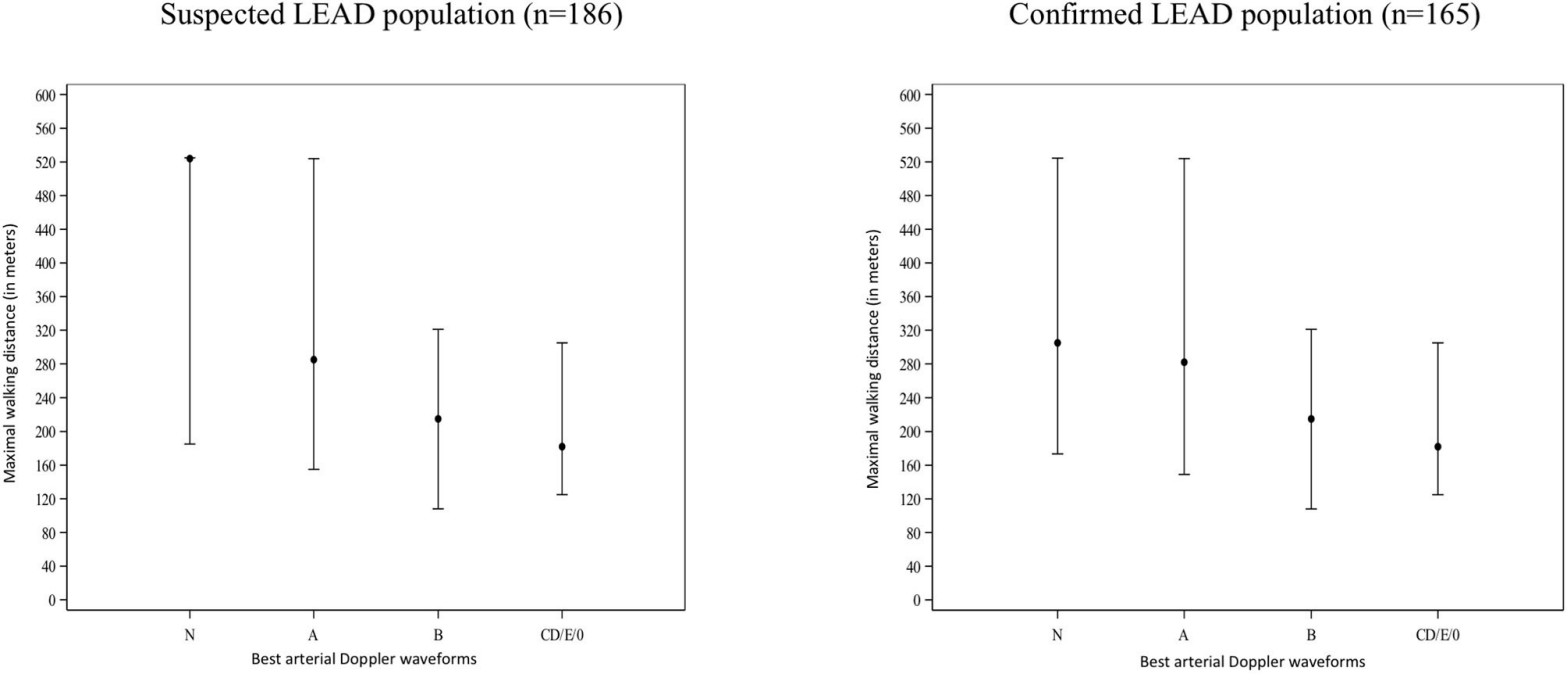
Maximal walking distance and arterial Doppler waveforms in patients suspected of LEAD and in confirmed LEAD. Results shown as medians [Q1 First quartile-Q3 Third quartile]. Simplified Saint-Bonnet classification sorts types according to severity of arterial lesions (Saint-Bonnet N, Saint-Bonnet A, Saint-Bonnet B, Saint-Bonnet CD, Saint-Bonnet E, Saint-Bonnet 0 [i.e., no flow]. Saint-Bonnet N and A are qualified as normal whereas, other types of flow are consistent with LEAD.

Median MWD from Doppler flow waveform categories are shown in Figure 3. In the suspected LEAD population, better arterial Doppler waveforms (i.e., closer to rank N) were associated with better walking distance (*p* = 0.0012). The relationship tended to be more marked with respect to the best arterial Doppler waveforms (*p* = 0.0012) compared with the worst arterial Doppler waveforms (*p* = 0.0088). A similar trend was found in confirmed LEAD patients yet no statistical significance was observed (best arterial waveform: *p* = 0.084; worst arterial waveform: *p* = 0.252).

### Relationship Between MWD and ABI

Median MWD in terms of ABI quartiles are shown in Table 2 for both populations. In the suspected LEAD population, MWD was significantly better in patients from the highest ABI quartile than in patients from the lowest (486 [206–524]m vs. 235 [118–335]m; *p* = 0.0006). Results of similar statistical significance were found in the confirmed LEAD population (354 [169;524]m vs. 235 [118;335]m; *p* = 0.048].

**Table 2:** Maximal walking distance according to Saint-Bonnet Classification and ABI.

	**Saint-Bonnet N**	**Saint-Bonnet A**	**Saint-Bonnet B**	**Saint-Bonnet CD/E/0**	
**SUSPECTED LEAD POPULATION (*****N*** **=** **186)**					
**Best arterial Doppler waveforms**					***p*****-value**
Maximal walking distance (m)	524 [185–525]	285 [155–524]	215 [108–321]	182 [125–305]	0.0012
**Worst arterial Doppler waveforms**					
Maximal walking distance (m)	525 [160–525]	304 [163–524]	223 [125–524]	189 [125–308]	0.0088
**Ankle–brachial index**					
	≥0.99	[0.84–0.99]	[0.64–0.84]	<0.64	
Maximal walking distance (m)	486 [206–524]	182 [103–357]	226 [131–368]	235[118–335]	0.006
Saint-Bonnet N	14 (29.2%)	5 (11.1%)	1 (2.0%)	1 (2.3%)	
Saint-Bonnet A	25 (52.1%)	23 (51.1%)	19 (38.8%)	0 (0.0%)	
Saint-Bonnet B	7 (14.6%)	13 (28.9%)	13 (26.5%)	11 (25.0%)	
Saint-Bonnet CD/E/0	2 (4.2%)	4 (8.9%)	16 (32.7%)	32 (72.7%)	
**CONFIRMED LEAD POPULATION** * **(*****N*** **=** **165)**					
**Best arterial Doppler waveforms**					***p*****-value**
Maximal walking distance (m)	305 [174–525]	282 [149–524]	215 [108–321]	182 [125–305]	0.084
**Worst arterial Doppler waveforms**					
Maximal walking distance (m)	347 [151–525]	236 [162–470]	223 [125–524]	189 [125–308]	0.252
**Ankle-Brachial index**					
	≥0.99	[0.84–0.99]	[0.64–0.84]	<0.64	
Maximal walking distance (m)	357 [169–524]	180 [103–357]	226 [131–368]	235 [118–335]	0.048
Saint-Bonnet N	7 (23.3%)	3 (7.1%)	1 (2.0%)	1 (2.3%)	
Saint-Bonnet A	14 (46.7%)	22 (52.4%)	19 (38.8%)	0 (0.0%)	
Saint-Bonnet B	7 (23.3%)	13 (31.0%)	13 (26.5%)	11 (25.0%)	
Saint-Bonnet CD/E/0	2 (6.7%)	4 (9.5%)	16 (32.7%)	32 (72.7%)	

LEAD means Lower Extremity Artery Disease. Results are presented median [Quartile 1; Quartile 3], or number of observation (%). *Criterion for Lower Extremity Artery Disease (LEAD) were Ankle brachial index ≤ 0.90 or abnormal Doppler waveforms or Post-exercise ABI decrease ≥18.5% or Delta from Rest Oxygen Pressure (DROP) ≤ -15 mmHg.

### Relationship Between ABI and Arterial Doppler Waveforms

**Figure 4:**
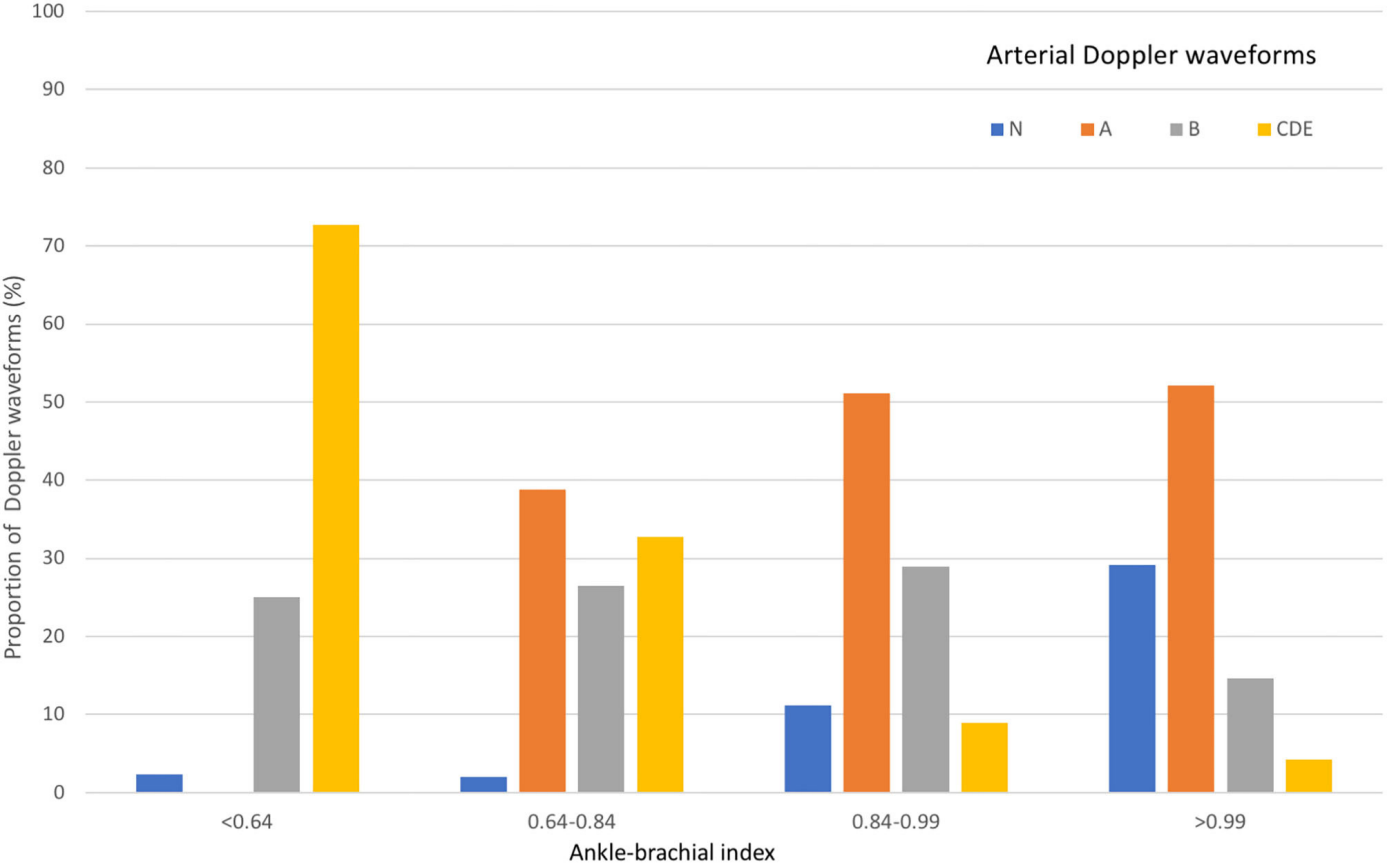
Distribution of best arterial Doppler waveforms based on ankle-brachial index.

In the suspected LEAD population, arterial Doppler waveform distribution in terms of ABI quartiles are shown in Figure 4. Abnormal arterial Doppler waveforms were found in 9 out of 48 patients (19%) with ABI ≥0.99 (regarded as normal ABI) whereas, normal arterial Doppler waveforms were observed in 21 (23%) patients with ABI <0.84 (Table 2). Patients from the highest ABI quartile had higher prevalence of Saint-Bonnet N and A lower limb extremity arterial waveforms than those from the lowest ABI quartile (*p* < 0.0001). A higher proportion of Saint-Bonnet CDE0 arterial Doppler waveforms were found in the lowest ABI quartile group compared with the highest ABI quartile group (*p* < 0.0001).

### Multivariate Analysis With Regard to Clinical Characteristics and Clinical Readings at Rest

Findings from univariate and multivariate analysis pertaining to patient characteristics and resting clinical readings (ABI and Doppler waveforms) are outlined in Table 3. In the multivariate model, arterial Doppler waveforms alone were associated with MWD (*p* = 0.0009) in the suspected LEAD population. Analysis of confirmed LEAD patients demonstrated a similar trend regarding arterial Doppler waveforms although no statistical significance was found (*p* = 0.083). There was no statistical association between ABI and MWD in the LEAD population (*p* = 0.323).

**Table 3:** Univariate and multivariate analysis findings including patient characteristics and clinical readings at rest (ABI and Doppler waveforms) in suspected LEAD population (*n* = 186).

**Variables**	**Univariate analysis**		**Multivariate analysis**	
	**Beta**	***p*-value**	**Beta**	***p*-value**
Gender				
Women vs. men	−8.094	0.4184		
Age	−0.422	0.1924		
Body mass index	−1.548	0.0752		
Diabetes vs. no diabetes	−22.56	0.0206		
Dyslipidemia vs. no dyslipidemia	−14.14	0.0950		
Hypertension vs. no hypertension	−12.39	0.1474		
Smoking status		0.5955		
Former vs. never	8.849			
Active smoker vs. never	0.823			
History of sleep apnea disorder vs. no history of sleep apnea disorder	4.798	0.6995		
History of myocardial infarction vs. no history	−14.46	0.0944		
Lower limb arterial stent vs. no history	−18.89	0.0294		
History of lower extremity vascular bypass	−6.460	0.5614		
Antiplatelets vs. none	−7.943	0.3824		
Statins vs. none	−11.32	0.1669		
Sartans vs. none	−7.162	0.4789		
Angiotension converting enzyme inhibitors vs. none	−11.06	0.1665		
Ankle-brachial index	30.947	0.0342		
Best arterial Doppler waveforms (reference CD/E/0)		0.0009		0.0009
Saint-Bonnet N	48.720		48.720	
Saint-Bonnet A	22.984		22.984	
Saint-Bonnet B	3.671		3.671	

### Multivariate Analysis in Terms of Clinical Characteristics, Resting Clinical Readings and Exercise Data

When exercise variables (post-exercise ABI or DROP) were added to the multivariate model, exercise variables alone were associated with MWD in the suspected LEAD population (*p* < 0.001 for post-exercise ABI and *p* < 0.001 for DROP) and in the confirmed LEAD population (*p* = 0.016 for post-exercise; *p* = 0.021 for DROP).

## Discussion

Clinical tests, such as ABI and arterial Doppler waveforms analysis are commonly used for LEAD diagnosis and follow-up. The results from the present study suggest that (i) arterial Doppler waveforms are independently associated with MWD in patients suspected of LEAD with exertional limb symptoms; and (ii) arterial Doppler waveforms tended to be associated with MWD in confirmed LEAD patients with exertional limb symptoms.

Clinicians are keen to have at their disposal the clinical wherewithal to determine functional impairment in patients with exertional limb symptoms so as to avoid squandering time on objective treadmill stress testing and make savings from a health policy perspective. To this end, several tools have been made available, such as questionnaires (27, 28), clinical tests (7, 9, 10), and regression equations based on clinical measurement (8, 29).

As previously mentioned, there has been relatively little consensus on any relationship between ABI and MWD in LEAD patients (7–11). Our study findings suggest that there is indeed a relationship between ABI and MWD in patients suspected of LEAD with exertional limb symptoms even when a single stage protocol is used to assess MWD (Strandness 10%; 3.2 km/h) but that any such relationship was not observed in confirmed LEAD patients. It is true that other studies in which a relationship between resting ABI and MWD was established used incremental protocols (8, 10). In our laboratory, a single stage protocol was employed because exercise TcPO_2_ was measured as part of the treadmill stress test and DROP has only been endorsed in relation to single stage protocols (17, 20, 22, 24).

As shown in Table 2, ABI is apparently a good predictor of walking impairment depending on whether it is normal or otherwise. When ABI is pathological, however, no correlation is observed between ABI and MWD (*p* > 0.05 regarding the bottom three quartiles). This likely stems from the fact that: (i) ABI can be affected by increased arterial stiffness in patients with diabetes or renal insufficiency (our study included 38 patients with diabetes and 10 with renal insufficiency for instance) (30–32); and (ii) resting ABI is by definition measured at rest and therefore does not reflect exercise hemodynamics.

According to the results of the present study, Doppler flow waveform analysis and categorization as per the Saint-Bonnet classification system is robust, exhibiting a trend that persists among waveforms found in the confirmed LEAD population (*n* = 165). However, altered arterial Doppler waveforms are not necessarily indicative of low MWD. As demonstrated (Table 2), even in the group with Saint-Bonnet B or CDE0, the third quartiles were high with MWD ranging from 524 to 308 m, respectively. ABI findings yielded a similar result. Poor ABI does not point to low MWD. Such lack of accuracy in resting clinical tests designed to predict MWD may have a physiological explanation. Collateral vessels are believed to enlarge during exercise in animals and humans (33).

Another point of interest arising from this study is that the relationship between Doppler flow waveforms and MWD appears stronger when the healthier symptomatic lower limb extremity artery is investigated (*p* = 0.0009 for the healthier artery vs. *p* = 0.008 for the more unhealthy artery). This suggests that MWD could primarily be restricted by the best lower limb arterial flow. There could be several reasons for this: (i) endothelial function in the less damaged artery may be better preserved, meaning that the endothelium-dependent vasodilation mechanisms that operate during exercise could still be present (34); (ii) there may be a more substantial increase in exercise-induced shear stress; and (iii) a collateral arterial network is more likely to arise from a less severely damaged artery. These reasons should incite physicians to consistently assess all limb arteries when evaluating LEAD patients.

As MWD was only assessed via a single stage protocol (10% slope, 3.2 km/h) in the present study, we cannot ascertain that similar results will be found using other protocols (35). However, it would be of particular benefit to assess the relationship between arterial Doppler waveforms and real-life MWD as assessed by GPS or other activity monitors (36, 37) given that real-life MWD and protocol MWD have been shown to differ significantly.

Furthermore, when exercise variables (post-exercise ABI and DROP) were incorporated into the model, these were the only variables that remained significant in both populations. Although not surprising in that post-exercise ABI and DROP reflect hemodynamic changes linked to exercise, this result emphasizes the strength of the relationship between Doppler flow waveform profiles and MWD in as much as only exercise variables seem to yield stronger association.

The heterogeneity of Doppler flow waveform characterization could explain why the prospect of a relationship between arterial Doppler waveforms and MWD has never been previously investigated. Classification systems are a very useful tool in improving the homogeneity of Doppler flow waveform characterization (38–40). The simplified Saint-Bonnet classification system was used in the present study as recommended by professors of the French College of Vascular Medicine (13, 19) the SFMV/SCVE consensus (14), and a study has shown that it yields superior categorization rates by comparison with other classification systems (41).

### Limitations

This is not a single-center study, and since our vascular laboratory physicians are highly trained to measure ABI with intra-observer coefficient of variation (CV) for resting ABI equal to 9.4% (typical estimation error is 0.06), and are familiar with the Saint-Bonnet system (19, 42), our results cannot be extrapolated to other operators. Furthermore, our patients' characteristics differ from other studies that have investigated the relationship between ABI and MWD with notably lower mean age and a smaller proportion of patients suffering from diabetes (7–11). Furthermore, our walking test protocol differs from other study protocols. Hence there is likely to be a discrepancy between the results obtained herein and those derived from other walking tests. A further limitation lies in the number of patients included in this study: several patients were excluded for lack of data and only 186 patients were reviewed. Even though this constitutes the one of the largest samples as compared to previous studies addressing the relationship between ABI and MWD, it is nonetheless a small sample (7–11). Moreover, analysis was conducted on two categories of patients: those suspected of LEAD based on treadmill stress test symptoms (i.e., claudication) and so-called “confirmed LEAD” patients based on treadmill stress test symptoms and at least one hemodynamic criterion for LEAD (either ABI or Doppler waveforms, or DROP or post-exercise ABI). It was important to analyze both categories since if only patients with LEAD based on ABI ≤ 0.90 had been investigated, patients with deceptively normal ABI readings would potentially have been excluded. Finally, on the subject of Doppler flow classification, the simplified Saint-Bonnet classification system is a relatively recent system with which many vascular physicians are unfamiliar. In the absence of E and 0 waveforms even greater simplification was required, rendering extrapolation of our results to those categories impossible.

## Conclusion

The results of the present study suggest that of the various resting clinical parameters used, Doppler flow waveforms categorized via simplified Saint-Bonnet classification are associated with MWD in patients suspected of LEAD. There is however, a stronger association between exercise variables (post-exercise ABI and DROP) and MWD.

## Data Availability Statement

The raw data supporting the conclusions of this article will be made available by the authors, without undue reservation.

## Ethics Statement

The studies involving human participants were reviewed and approved by institutional review board (IRB; no. 17.12). The patients/participants provided their written informed consent to participate in this study.

## Author Contributions

AL and GM: protocol conception and design. AMi, QT, AG, AC, AMé, and GM: data acquisition. AMi, AL, DL, GM, and EL: data analysis and writing of paper. AMi and GM: data interpretation and drafting of paper. Each author revised the report and approved the submitted version of the manuscript. Each author has agreed both to be personally accountable for the his/her own contribution and to ensure that questions related to the accuracy or integrity of any part of the work, even those in which the author was not personally involved, are appropriately investigated, resolved, and the resolved outcome documented in the literature. All authors contributed to the article and approved the submitted version.

## Conflict of Interest

The authors declare that the research was conducted in the absence of any commercial or financial relationships that could be construed as a potential conflict of interest.
