# Corrigendum: Arterial Doppler Waveforms Are Independently Associated With Maximal Walking Distance in Suspected Peripheral Artery Disease Patients

**DOI:** 10.3389/fcvm.2021.699346

**Published:** 2021-05-13

**Authors:** Annaïg Miossec, Quentin Tollenaere, Damien Lanéelle, Antoine Guilcher, Antoine Métairie, Estelle Le Pabic, Awenig Carel, Alexis Le Faucheur, Guillaume Mahé

**Affiliations:** ^1^Vascular Medicine Unit, CHU Rennes, Rennes, France; ^2^Vascular Medicine Unit, CHU Caen Normandie, Caen, France; ^3^CHU Rennes, Inserm, CIC 1414 (Clinical Investigation Center), Rennes, France; ^4^Univ Rennes, M2S-EA 7470, Rennes, France; ^5^Univ Rennes 1, Rennes, France

**Keywords:** Doppler waveforms, peripheral artery disease, maximal walking distance, ankle-, brachial index, peripheral artery (occlusive) disease

In the original article, there was a mistake in Table 2 as published. The following subtitle **“Best arterial Doppler waveforms”** was missing in the **“Confirmed LEAD population (*n*** **=** **165)”**. The corrected Table 2 appears below.

**Table 2:** Maximal walking distance according to Saint-Bonnet Classification and ABI.

	**Saint-Bonnet N**	**Saint-Bonnet A**	**Saint-Bonnet B**	**Saint-Bonnet CD/E/0**	
**SUSPECTED LEAD POPULATION (*****N*** **=** **186)**					
**Best arterial Doppler waveforms**					***p*****-value**
Maximal walking distance (m)	524 [185–525]	285 [155–524]	215 [108–321]	182 [125–305]	0.0012
**Worst arterial Doppler waveforms**					
Maximal walking distance (m)	525 [160–525]	304 [163–524]	223 [125–524]	189 [125–308]	0.0088
**Ankle–brachial index**					
	≥0.99	[0.84–0.99]	[0.64–0.84]	<0.64	
Maximal walking distance (m)	486 [206–524]	182 [103–357]	226 [131–368]	235[118–335]	0.006
Saint-Bonnet N	14 (29.2%)	5 (11.1%)	1 (2.0%)	1 (2.3%)	
Saint-Bonnet A	25 (52.1%)	23 (51.1%)	19 (38.8%)	0 (0.0%)	
Saint-Bonnet B	7 (14.6%)	13 (28.9%)	13 (26.5%)	11 (25.0%)	
Saint-Bonnet CD/E/0	2 (4.2%)	4 (8.9%)	16 (32.7%)	32 (72.7%)	
**CONFIRMED LEAD POPULATION** * **(*****N*** **=** **165)**					
**Best arterial Doppler waveforms**					***p*****-value**
Maximal walking distance (m)	305 [174–525]	282 [149–524]	215 [108–321]	182 [125–305]	0.084
**Worst arterial Doppler waveforms**					
Maximal walking distance (m)	347 [151–525]	236 [162–470]	223 [125–524]	189 [125–308]	0.252
**Ankle-Brachial index**					
	≥0.99	[0.84–0.99]	[0.64–0.84]	<0.64	
Maximal walking distance (m)	357 [169–524]	180 [103–357]	226 [131–368]	235 [118–335]	0.048
Saint-Bonnet N	7 (23.3%)	3 (7.1%)	1 (2.0%)	1 (2.3%)	
Saint-Bonnet A	14 (46.7%)	22 (52.4%)	19 (38.8%)	0 (0.0%)	
Saint-Bonnet B	7 (23.3%)	13 (31.0%)	13 (26.5%)	11 (25.0%)	
Saint-Bonnet CD/E/0	2 (6.7%)	4 (9.5%)	16 (32.7%)	32 (72.7%)	

LEAD means Lower Extremity Artery Disease. Results are presented median [Quartile 1; Quartile 3], or number of observation (%). *Criterion for Lower Extremity Artery Disease (LEAD) were Ankle brachial index ≤ 0.90 or abnormal Doppler waveforms or Post-exercise ABI decrease ≥18.5% or Delta from Rest Oxygen Pressure (DROP) ≤ -15 mmHg.

The authors apologize for this error and state that this does not change the scientific conclusions of the article in any way. The original article has been updated.

